# Supplementary material for: Modeling common Alzheimer’s disease with high and low polygenic risk in human iPSC: A large-scale research resource
Source: Stem Cell Reports. 2025 Jul 3;20(8):102570. doi: 10.1016/j.stemcr.2025.102570 (PMC12365843; doi:10.1016/j.stemcr.2025.102570)
Supplement: Document S1. Figure S1, Tables S1–S3, and supplemental methods [file mmc1.pdf]

Stem Cell Reports, Volume *20*

Supplemental Information

Modeling common Alzheimer's disease with high and low polygenic risk in human iPSC: A large-scale research resource

Emily Maguire, Jincy Winston, Sarah H. Ellwood, Rachel O'Donoghue, Bethany Shaw, Atahualpa Castillo Morales, Samuel Keat, Alexandra Evans, Rachel Marshall, Lauren Luckcuck, Laura Brown, Elisa Salis, Ganna Leonenko, Nicola Denning, EADB consortium, Nicholas D. Allen, Valentina Escott-Price, Caleb Webber, Philip R. Taylor, Rebecca Sims, Sally A. Cowley, Julie Williams, Sarah M. Carpanini, and Hazel Hall-Roberts

Supplemental figures

A Mycoplasma

Sample	Reading 1	Reading 2	Ratio	Status
Positive control	3.113	137.3	44.1	Positive
Negative control	4.135	0.713	0.17	Negative
DRICUi011-A	2.048	0.953	0.47	Negative

B CNV correlation between DRICUi011-A iPSC and donor PBMC

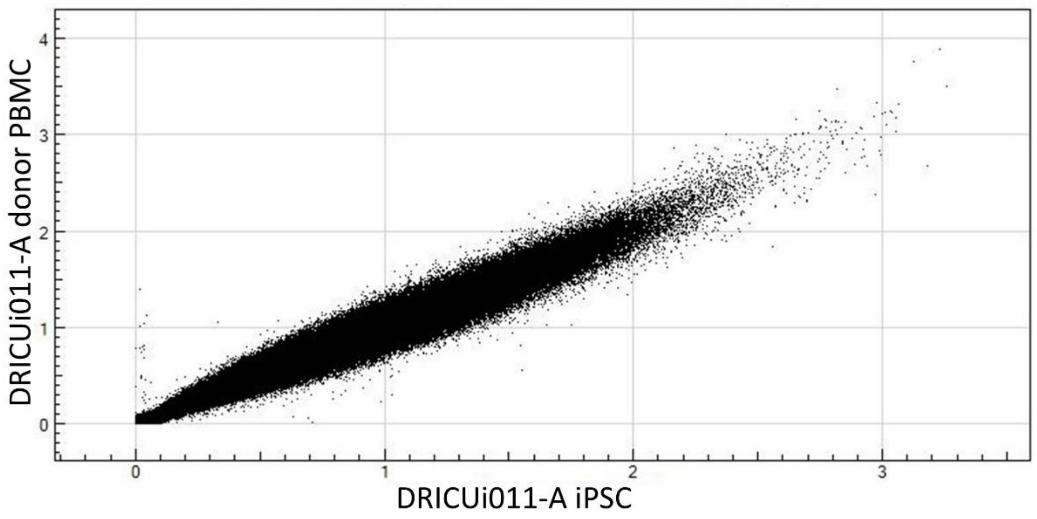

Figure S1. A) DRICUi011-A iPSC test negative for mycoplasma at passage 14, ratios between 0-0.999 indicate a negative result (green), ratios above 1.3 indicate a positive result (red). B) Copy number variant analysis shows a strong correlation between iPSC-line DRICUi011-A and the donor PBMC for that line, regression coefficient R^2= 0.98.

Supplemental tables

Table S1: Primers used to identify clearance of Sendai virus.

Marker	Forward primer	Reverse primer	Product size
**SeV**	GGATCACTAGGTGATATCGAG	ACCAGACAAGAGTTTAAGAGATATGTATC	181bp
**KOS**	ATGCACCGCTACGACGTGAGCGC	ACCTTGACAATCCTGATGTGG	528bp
**KLF4**	TTCCTGCATGCCAGAGGAGCCC	AATGTATCGAAGGTGCTCAA	410bp
**SeV-Myc**	AACTGACTAGCAGGCTTGTCG	TCCACATACAGTCCTGGATGATGATG	532bp
**β-Actin**	AGGCATCCTCACCCTGAAG	TCCATGCCCAGGAAGGAAG	632bp

SeV = Sendai virus, KLF4 = Krüppel-like factor 4 (Klf4), KOS = KLF4, Octamer-binding transcription factor 3/4 (Oct3/4), & Sex determining region Y-box 2 (Sox2), bp = base pairs.

Table S2: Details regarding antibodies used for flow cytometry of pluripotency markers.

Antibody	Supplier	Catalogue number	Stock concentration	Staining concentration
**Alexa Fluor® 488 Mouse IgM, κ Isotype control**	Biolegend	401617	500 µg/mL	3 µg/mL
**Alexa Fluor® 488 anti-human TRA-1-60-R**	Biolegend	330614	150 µg/mL	3 µg/mL
**Rabbit (DA1E) mAb IgG XP® Isotype Control (Alexa Fluor® 647 Conjugate)**	Cell Signaling Technology	2985S	100 µg/mL	0.67 µg/mL
**Nanog (D73G4) XP® Rabbit mAb (Alexa Fluor® 647 Conjugate)**	Cell Signaling Technology	5448S	50 µg/mL	0.67 µg/mL

Table S3: Details regarding antibodies used for immunocytochemistry staining for trilineage markers.

Germ Layer	Antibody	Code/ Company	Against	Stock concentration	Staining concentration
**Ectoderm**	OTX2	AF1979	Goat	0.2 mg/mL	10 µg/mL
	PAX6	901302	Rabbit	2 mg/mL	40 µg/mL
**Mesoderm**	Brachyury	AF2085	Goat	0.2 mg/mL	10 µg/mL
	CXCR4	MAB172	Mouse	0.5 mg/mL	8 µg/mL
**Endoderm**	SOX17	AF1924	Goat	0.2 mg/mL	10 µg/mL
	CXCR4	MAB172	Mouse	0.5 mg/mL	8 µg/mL

Supplemental methods

Cohort assessment for AD

The collection of the samples was through multiple channels, including specialist NHS services and clinics, research registers and Join Dementia Research (JDR) platform. The participants were assessed at home or in research clinics along with an informant, usually a spouse, family member or close friend, who provided information about and on behalf of the individual with dementia. Established measures, validated for AD by Holmes et al., [1] were used to ascertain clinical diagnosis and the disease severity: CAMDEX, Mattis Dementia Rating Scale, Mini-Mental state examination (MMSE), Bristol activities of daily living (BADL), Clinical Dementia Rating scale (CDR), Neuropsychiatric Inventory (NPI), Global Deterioration Scale (GiDS), Addenbrooke's Cognitive Examination (ACE-r), Geriatric Depression Scale (GeDS) and National Adult Reading Test (NART). All AD cases met criteria for either probable (NINCDS-ADRDA, DSM-IV) or definite (CERAD) AD. Control participants were recruited from GP surgeries and by means of self-referral. All elderly controls were screened for dementia using the Mini Mental State Examination (MMSE) or ADAS-cog. Control samples were chosen to match case samples for sex and ethnicity.

Informed consent for AD subjects

Capacity to consent was assessed by a trained, experienced research assistant at all visits. In the case where an individual lacked capacity to consent, an appropriate consultee was identified in accordance with section 32 of the Mental Capacity Act (2005). All participants and consultees received verbal and written information about the study and had the opportunity to ask questions. Participation was voluntary, with the option to withdraw consent at any time, without giving a reason. Any signs of the participant objecting or feeling distressed during a visit were assumed as refusal of consent, leading to cessation of sample/data collection.

Data privacy

The confidentiality of participants in this study is preserved in accordance with the Data Protection Act (1998). All participant information is stored in a linked pseudo-anonymous format, with participants assigned an ID code. Personal identifying information linked to the ID code is stored separately from the clinical and genetic data in a password-protected spreadsheet, within Cardiff University's data security infrastructure, and can only be accessed by designated team members.

Quality control of Genetic data

Our PRS based prioritisation of cell lines was based on genotyping data derived from multiple DRI Cardiff collections. Genotyping of carried out in different stages and funded by MRC and CADR grant applications.

a) GERAD dataset (3332 cases and 7355 controls) was genotyped on the Illumina550 array (GRCh37/hg19), with Quality-Control (QC) analysis described in previously [2]. Further it was imputed via Michigan Imputation server using Minimac3 [3] and Haplotype Reference Consortium (HRC)3 reference panel. After imputation QC: variants were excluded with minor allele frequency (MAF) <1%, missing data proportion (MISS) >5% and Hardy-Weinberg Equilibrium (HWE) ($p \leq 10^{-6}$) and poor imputation INFO score of <0.7. Illumina550 array did not have APOE gene and APOE genotyping was done separately.

b) EADB 2 batches were genotyped on Illumina GSA (GRCh37/hg19) array in Lille (5679 samples and 678,826 SNPs. Sample QC: exclude gender mismatch (164 individuals); duplicates in name (3 individuals), het >+-0.1 (11 individuals); population stratification (42 individuals). Variant QC: missingness >2%, MAF<1%, (HWE) ($p \leq 10^{-6}$). Leaving 5283 samples on 491611 samples. APOE genotype was performed separately. This dataset was part of EADB consortium.

c) 1909 individuals were genotyped on Illumina GSA array (GRCh37/hg19) covering 665,608 variants. APOE genotype was included as part of the array. This data is independent from IGAP and EADB consortiums.

Datasets b) and c) were combined together and additional quality control performed to exclude samples with high heterozygosity (Het ± 0.05); individual missingness > 3%; high identity by descent (pi^hat > 0.2) or gender mismatch. Variants with MAF < 1%; missingness > 5%; and that break Hardy-Weinberg Equilibrium (HEW $p \leq 10^{-6}$). Furthermore, control-control analysis was performed and variants were excluded with $p<5x10^{-3}$, leaving 6,870 samples and 480,021 variants. This combined data set was then imputed using Michigan Imputation server's Minimac3[3] and Haplotype Reference Consortium (HRC)3 reference panel. Following imputation, variants with minor allele frequency (MAF) <1%, missing data proportion (MISS) >5% and Hardy-Weinberg Equilibrium (HWE) ($p \leq 10^{-6}$) and poor imputation INFO score of <0.7 were excluded, leaving 7,518246 polymorphisms. Finally, all the datasets were combined together with genotyping data from non-AD affected individuals from the 1958 National Child Development (N= 4,032) [4] as a population control, for the purposes of PRS standardisation and principal components were re-calculated.

Primary PRS calculation (C + T)

For the PRS calculation we used the summary statistics from the largest available clinically assessed case-control GWAS study on AD [5] ($N = 63,926$) to generate genetic scores for all participants in the cohorts described above as the weighted sum of the risk alleles. PRS were generated using PRSice-2 using default P-value thresholds. PRSice-2 utilizes the most common approach for PRS calculation of clumping and thresholding (C + T), where markers most strongly associated with the phenotype of interest are preferentially retained. Clumping was performed using an LD r^2=0.1 and a window size of 1000 kb. PRS.no.APOE was calculated excluding the *APOE* region (chromosome 19:44.4–46.5 Mb) due to the high LD in this region. PRS.AD was calculated as a weighted sum of PRS.no.APOE and *APOE*(ε2 + ε4), where *APOE* effects were weighted with effect sizes (B(ε2) = −0.47 and B(ε4) = 1.12) [6]. This means that, for APOE ε3 homozygous individuals, their PRS.AD is equivalent to their PRS.no.APOE. PRS.AD was then adjusted by regressing against 8 principal components and then standardised within the sample. Subsequently, PRS.AD of all of the individuals in our cohorts were standardised against the mean and standard deviation of the PRS.AD of the population controls in 1958BC.

Commercial use

Consent provisions and study ethics permit commercial use of the iPSC and related data. However, the iPSC were generated using CytoTune technology and are subject to the terms of Limited use label License No: 518 CytoTune™ Technology for Products, as detailed below.

Notice to Purchaser: This product is authorized for reprogramming methods that involve or pertain to the preparation of iPS cells or related cells. The purchase of this product conveys to the purchaser the limited, non-transferable right to use the purchased amount of product to perform internal use and for educational purposes. No right to resell this product or any of its components, or iPS cells or related cells generated by use of the product, or derivatives thereof (hereafter "the Materials") is conveyed expressly, by implication, or by estoppel. For clarity, purchasers have the right to use third party service providers for generating iPS cells and derivatives for the benefit of such purchasers. Purchasers can deposit the Materials with not-for-profit repositories ("Repositories") and transfer cells to not-for-profit research entities (not affiliated with a for-profit organization) for their internal research. Such recipient Repositories and not-for-profit research entities are allowed to distribute the Materials not-for financial gain to other users for their internal research. If the Materials are transferred to other users in accordance with the terms of this label license accompanying the product (hereafter "Label License"), the transferring party should notify recipients of such Materials of these terms by transferring a copy of the Label License to the recipients.

To obtain commercial rights for the sale of the Materials or for a fee-for-service generation of the Materials other than as allowed in paragraph 1 above, purchasers are requested to contact DNAVEC Corporation at cytotune@dnavec-corp.com. For all other commercial applications relating to the use of the Materials, purchasers might be required to contact iPS Academia Japan. Customers may contact iPS Academia Japan either directly at license@ips-ac.co.jp or through DNAVEC Corporation.

Supplemental references

1. Holmes, C., Cairns, N., Lantos, P. L. & Mann, A. Validity of current clinical criteria for Alzheimer's disease, vascular dementia and dementia with Lewy bodies. *British Journal of Psychiatry* **174**, 45–50 (1999).

2. Harold, D. *et al.* Genome-wide association study identifies variants at CLU and PICALM associated with Alzheimer's disease. *Nat Genet* **41**, 1088–1093 (2009).

3. Das, S. *et al.* Next-generation genotype imputation service and methods. *Nat Genet* **48**, 1284–1287 (2016).

4. University College London, UCL Social Research Institute, C. for L. S. National Child Development Study [data series]. *UK Data Service 14th release* (2024) doi:http://doi.org/10.5255/UKDA-Series-2000032.

5. Kunkle, B. W. *et al.* Genetic meta-analysis of diagnosed Alzheimer's disease identifies new risk loci and implicates Aβ, tau, immunity and lipid processing. *Nat Genet* **51**, 414–430 (2019).

6. Leonenko, G. *et al.* Identifying individuals with high risk of Alzheimer's disease using polygenic risk scores. *Nat Commun* **12**, 4506 (2021).
