# Supplementary material for: Modeling common Alzheimer’s disease with high and low polygenic risk in human iPSC: A large-scale research resource
Source: Stem Cell Reports. 2025 Jul 3;20(8):102570. doi: 10.1016/j.stemcr.2025.102570 (PMC12365843; doi:10.1016/j.stemcr.2025.102570)
Supplement: Document S2. Article plus supplemental information [file mmc2.pdf]

Stem Cell Reports
Resource
OPEN ACCESS

Modeling common Alzheimer's disease with high and low polygenic risk in human iPSC: A large-scale research resource

Emily Maguire,[1,5] Jincy Winston,[1,5] Sarah H. Ellwood,[2,5] Rachel O'Donoghue,[1,5] Bethany Shaw,[1] Atahualpa Castillo Morales,[1] Samuel Keat,[1] Alexandra Evans,[3] Rachel Marshall,[3] Lauren Luckcuck,[1] Laura Brown,[2] Elisa Salis,[1] Ganna Leonenko,[1] Nicola Denning,[1] EADB consortium, Nicholas D. Allen,[4] Valentina Escott-Price,[1,3] Caleb Webber,[1] Philip R. Taylor,[1] Rebecca Sims,[3] Sally A. Cowley,[2] Julie Williams,[1,6,*] Sarah M. Carpanini,[1,6,*] and Hazel Hall-Roberts[1,6,7,*]
[1]UK Dementia Research Institute at Cardiff University, Maindy Road, CF24 4HQ Cardiff, UK
[2]James and Lillian Martin Centre for Stem Cell Research, Sir William Dunn School of Pathology, University of Oxford, South Parks Road, OX1 3RE Oxford, UK
[3]Division of Psychological Medicine and Clinical Neurosciences, Cardiff University, Maindy Road, CF24 4HQ Cardiff, UK
[4]School of Biosciences, Cardiff University, Museum Avenue, CF10 3AX Cardiff, UK
[5]These authors contributed equally
[6]Senior author
[7]Lead contact
*Correspondence: williamsj@cardiff.ac.uk (J.W.), carpaninis@cardiff.ac.uk (S.M.C.), hall-robertsh@cardiff.ac.uk (H.H.-R.)
https://doi.org/10.1016/j.stemcr.2025.102570

SUMMARY

Common forms of Alzheimer's disease (AD) are complex and polygenic. We have created a research resource that seeks to capture the extremes of polygenic risk in a collection of human induced pluripotent stem cell (iPSC) lines from over 100 donors: the IPMAR Resource (iPSC Platform to Model Alzheimer's Disease Risk). Donors were selected from a large UK cohort of 6,000+ research-diagnosed early or late-onset AD cases and elderly cognitively healthy controls, many of whom have lived through the age of risk for disease development (>85 years). We include iPSC with extremes of global AD polygenic risk (high-risk late-onset AD: 34; high-risk early-onset AD: 29; low-risk control: 27) as well as those reflecting complement pathway-specific genetic risk (high-risk AD: 9; low-risk controls: 10). All iPSC have associated clinical, longitudinal, and genetic datasets and will be available through collaboration or from cell (EBiSC) and data (DPUK) repositories.

INTRODUCTION

Alzheimer's disease (AD) is a progressive neurodegenerative disorder characterized by cognitive decline, memory loss, and impaired daily functioning. At the core of Alzheimer's pathology is the accumulation of β-amyloid plaques and neurofibrillary tangles composed of hyperphosphorylated tau in the brain and the degeneration and death of neurons (Serrano-Pozo et al., 2011).

AD is broadly categorized into early onset (EOAD, ∼5% of cases), occurring before the age of 65, and late onset (LOAD, ∼95% of cases), typically manifesting after 65 (Reitz et al., 2020). Both categories appear to have a strong genetic component, with heritability estimates between 92% and 100% for EOAD and 60% and 80% for LOAD (Wingo et al., 2012). Within EOAD, approximately 10% of cases occur as a result of causal fully penetrant mutations in the genes encoding either amyloid precursor protein or presenilin 1 and 2 (Wingo et al., 2012); this form of disease is often referred to as familial AD (FAD). This can be contrasted with sporadic or "common" AD (EOAD and LOAD), which has no known autosomal-dominant cause and is contributed to by a combination of genetic and environmental risk factors. The *apolipoprotein ε4* allele (*APOE ε4*) is the most penetrant genetic risk variant for common AD, with reported odds ratio between 3.62 and 34.3, depending on the population examined (Belloy et al., 2023; Kukull et al., 1996; Saddiki et al., 2020). In addition to *APOE ε4*, common AD heritability has been associated with >70 common and rare genetic variants, identified by genome-wide association studies (GWASs) (Bellenguez et al., 2022). These genetic variants implicate roles for immune responses, complement, endocytosis, and lipid transport in AD pathogenesis, in addition to amyloid-β and tau processing (Sims et al., 2020).

Researchers can use a technique called polygenic risk score (PRS) analysis to quantify an individual's genetic susceptibility to developing common AD. PRS aggregates information from all identified genetic variants known to impact the overall risk of developing AD. By assessing an individual's genetic profile and summing up the effects of these variants, a PRS value provides a personalized estimate of an individual's likelihood of developing AD. While not a definitive predictor, the PRS offers valuable insights into the genetic component of Alzheimer's and is able to predict an individual's risk of developing the disease with 84% accuracy (Escott-Price et al., 2017).

Various models are employed to explore the mechanisms of AD, each with its own set of advantages and limitations. This includes *in vivo* and *in vitro* work using mouse models

Published by Elsevier Inc. on behalf of International Society for Stem Cell Research.
This is an open access article under the CC BY license (http://creativecommons.org/licenses/by/4.0/).

and induced pluripotent stem cell (iPSC)-derived models containing autosomal-dominant mutations in genes associated with FAD (Drummond and Wisniewski, 2017; McKean et al., 2021; Penney et al., 2020; Raska et al., 2021). While these studies have allowed for many advances in our understanding of AD, in humans, there is significantly more severe neurofibrillary tangle formation and cerebral amyloid angiopathy in FAD than common AD, suggesting differences in the disease course (Ringman et al., 2016). Moreover, various crucial differences exist between mice and humans, which can limit translation of findings to human patients, including numerous differences in innate immune responses, known to be important in AD development (Drummond and Wisniewski, 2017; Franco Bocanegra et al., 2018; Mancuso et al., 2019). Other AD models have been engineered to contain single disease-associated single-nucleotide polymorphisms (SNPs) uncovered in genome-wide associated studies of common AD (Ganesan et al., 2024; Tran et al., 2023). While providing researchers with insight into specific pathways affected in AD, these models ignore the complexity and diversity of the genetic architecture of common AD (Sims et al., 2020).

Use of patient-derived iPSC is transforming many aspects of disease research. These iPSC can be generated by reprogramming patient tissues (e.g., blood or skin), prior to differentiation into any cell type of interest (Al Abbar et al., 2020). When using patient-derived iPSC to study common AD, it is crucial to consider the substantial genetic variation between individual donors (Sims et al., 2020). These variations are often unrelated to the AD phenotype, necessitating a large sample size to accurately distinguish differences between patient and control-derived cell lines and mitigate the noise introduced by these genetic variants (Sims et al., 2020). However, the fact that patient-derived iPSC retain the unique genetic makeup of the individual also presents as a huge advantage for researchers who wish to accurately represent and understand the common AD phenotype, as it allows researchers to model the genetic contribution to AD, and in particular the polygenicity of common AD (Sims et al., 2020). Existing large iPSC resources using common AD donors do not select donors based on their polygenic risk for the disease (Kondo et al., 2022; Lagomarsino et al., 2021). In order to accurately dissect the effect of common AD on cell function, it is important to utilize iPSC resources in which donors have been selected based on both their disease status and underlying genetic predisposition. It is evident that individuals with common AD each possess a unique set of genetic variants that influence disease risk. Therefore, by utilizing a large cohort of genetically informed samples, researchers can stratify individuals based on their specific genetic risk profiles. This stratification, when combined with data from functional cell assays or patient symptom analyses, could enable the identification of different underlying molecular dysfunctions within different patient cohorts. Consequently, this approach paves the way for personalized medicine in AD.

Here, we have selected and generated 109 iPSC lines: 63 from patients with common AD with high global PRS (including 34 from patients with LOAD and 29 from patients with EOAD), 27 from age-matched healthy controls with a low global PRS for common AD, 9 from patients with common AD with high complement pathway-specific PRS (henceforth termed “complement PRS”), and 10 from healthy controls with low complement PRS. We have named this iPSC resource IPMAR (iPSC Platform to Model Alzheimer’s disease Risk) as a major new iPSC resource to capture both high and low polygenic risk for common AD, alongside knowledge of diagnosed AD vs. control status. Large iPSC resources such as this one provide a comprehensive and patient-reflective disease model for drug screening that cannot be achieved using small numbers of FAD or common AD lines, which do not reflect the substantial genetic diversity underlying AD (Sims et al., 2020). Furthermore, this invaluable resource can be used to explore molecular and cellular mechanisms underlying common AD. Given the aforementioned advantages of IPMAR, our resource has potential to facilitate the identification of potential AD therapeutics with widespread clinical relevance.

RESULTS

Sample selection and generation

Donors were selected from participants within the Alzheimer’s Disease Cardiff Cohort (ADCC) using the aforementioned criteria. Following identification, cryopreserved peripheral blood mononuclear cells (PBMCs) were used to generate iPSC lines from the samples, with recent (“fresh”) donations prioritized, as detailed in Tables 1 and 2. 109 iPSC lines were generated in total. This comprised 90 lines selected with extremes of global AD PRS: 34 from LOAD donors (mean AD PRS 2.2 ± 0.5 SD, age of onset 72 ± 6 SD, 56% female), 29 from EOAD donors (mean AD PRS 2.1 ± 0.4 SD, age of onset 51 ± 3 SD, 55% female), and 27 from cognitively healthy controls (mean AD PRS -1.9 ± 0.4 SD, 59% female). For more information on cell line donors used for global AD PRS iPSC, see Table 1. Also included were 19 lines selected with extremes of complement PRS: 9 from LOAD donors (mean complement PRS 2.4 ± 0.3 SD, age of onset 71 ± 6, 78% female) and 10 from cognitively healthy controls (mean complement PRS −1.9 ± 0.2 SD, 70% female). For more information on cell line donors used for complement PRS iPSC, see Table 1. Within this paper, the line DRICUi011-A is used

Table 1. Cell line donor information for global AD PRS iPSC

Cell line ID	Status	Sex	AAO	AAI	APOE ε-alleles	AD PRS	Fresh/stored	Availability
DRICUi002-A	LOAD	F	70	96	33	2.00	stored	EBiSC
DRICUi003-A	EOAD	F	58	68	44	2.52	stored	EBiSC
DRICUi004-A	LOAD	F	68	71	44	3.06	stored	EBiSC
DRICUi005-B	LOAD	F	68	74	44	3.23	stored	EBiSC[a]
DRICUi006-A	LOAD	M	77	79	33	1.84	stored	EBiSC
DRICUi007-A	LOAD	M	70	74	34	2.08	stored	EBiSC
DRICUi008-A	control	M	N/A	88	33	−1.68	stored	EBiSC[a]
DRICUi009-B	control	F	N/A	80	33	−1.24	stored	EBiSC[a]
DRICUi010-A	LOAD	F	68	77	33	2.17	fresh	EBiSC
DRICUi011-A	LOAD	M	70	80	33	2.66	fresh	EBiSC
DRICUi012-A	LOAD	F	67	69	34	2.20	fresh	EBiSC
DRICUi013-A	LOAD	M	67	76	44	2.81	fresh	EBiSC
DRICUi014-A	control	F	N/A	89	33	−1.68	fresh	EBiSC
DRICUi015-A	control	F	N/A	95	33	−1.03	fresh	EBiSC[a]
DRICUi016-A	LOAD	F	66	71	33	2.83	fresh	EBiSC
DRICUi017-A	LOAD	F	68	74	34	2.40	fresh	EBiSC
DRICUi018-A	LOAD	M	67	78	44	2.63	fresh	EBiSC
DRICUi019-A	LOAD	F	66	76	44	3.20	fresh	EBiSC[a]
DRICUi020-A	control	M	N/A	83	33	−1.77	fresh	EBiSC[a]
DRICUi021-A	control	F	N/A	73	33	−2.51	fresh	EBiSC[a]
DRICUi022-A	control	F	N/A	80	33	−1.50	fresh	EBiSC
DRICUi023-A	LOAD	M	68	77	33	2.33	fresh	EBiSC
DRICUi024-A	LOAD	F	84	88	34	2.85	stored	EBiSC[a]
DRICUi025-A	control	M	N/A	82	33	−2.61	fresh	EBiSC
DRICUi026-A	control	M	N/A	92	33	−1.69	fresh	EBiSC
DRICUi027-A	LOAD	M	75	80	34	2.07	stored	EBiSC
DRICUi028-A	LOAD	M	68	72	34	2.44	stored	EBiSC
DRICUi029-A	LOAD	F	69	74	34	2.21	stored	EBiSC
DRICUi030-A	control	F	N/A	76	33	−2.27	stored	EBiSC[a]
DRICUi031-A	LOAD	F	79	81	33	2.29	stored	EBiSC[a]
DRICUi032-A	LOAD	M	74	78	34	2.26	stored	EBiSC[a]
DRICUi033-A	LOAD	M	66	71	33	1.25	fresh	EBiSC[a]
DRICUi034-A	LOAD	F	81	83	34	2.25	stored	EBiSC[a]
DRICUi036-A	LOAD	F	70	74	34	2.21	stored	EBiSC[a]

(*Continued on next page*)

Table 1. *Continued*

Cell line ID	Status	Sex	AAO	AAI	APOE ε-alleles	AD PRS	Fresh/stored	Availability
DRICUi037-A	control	F	N/A	77	33	−1.43	stored	EBiSC[a]
DRICUi038-A	control	M	N/A	76	33	−2.83	stored	EBiSC[a]
DRICUi039-A	control	M	N/A	71	33	−1.41	stored	EBiSC[a]
DRICUi040-A	control	M	N/A	79	33	−1.91	stored	EBiSC[a]
DRICUi041-A	LOAD	F	89	93	33	1.83	stored	EBiSC[a]
DRICUi042-A	LOAD	F	74	80	33	1.76	stored	EBiSC[a]
DRICUi043-A	LOAD	F	78	96	33	2.28	stored	EBiSC[a]
DRICUi044-A	control	M	N/A	73	33	−1.52	stored	EBiSC[a]
DRICUi045-A	LOAD	M	78	79	33	1.38	stored	EBiSC[a]
DRICUi046-A	control	F	N/A	73	33	−1.44	stored	EBiSC[a]
DRICUi047-A	LOAD	F	70	72	33	1.84	stored	EBiSC[a]
DRICUi048-A	LOAD	M	70	76	33	1.86	stored	on request[a]
DRICUi049-A	LOAD	M	66	71	33	1.76	stored	EBiSC[a]
DRICUi050-A	LOAD	M	68	72	33	1.78	stored	EBiSC[a]
DRICUi051-A	control	F	N/A	81	33	−1.85	stored	on request[a]
DRICUi052-A	control	M	N/A	81	33	−2.16	stored	EBiSC[a]
DRICUi053-A	LOAD	F	74	82	33	1.80	stored	EBiSC[a]
DRICUi054-A	LOAD	F	79	84	33	1.78	stored	EBiSC[a]
DRICUi055-A	LOAD	M	83	86	34	1.86	stored	EBiSC[a]
DRICUi056-A	EOAD	F	53	57	33	1.95	stored	on request[a]
DRICUi057-A	EOAD	M	48	53	33	1.94	stored	on request[a]
DRICUi058-A	EOAD	F	51	56	34	2.08	stored	on request[a]
DRICUi059-A	EOAD	F	52	55	33	1.94	stored	on request[a]
DRICUi060-A	EOAD	F	54	60	33	1.85	stored	on request[a]
DRICUi061-A	EOAD	M	51	57	33	1.80	stored	on request[b]
DRICUi062-A	EOAD	M	54	57	33	1.73	stored	on request[a]
DRICUi063-A	EOAD	F	47	53	34	2.06	stored	on request[b]
DRICUi064-A	EOAD	F	53	59	33	3.03	stored	on request[a]
DRICUi065-A	EOAD	F	50	56	33	2.55	stored	on request[b]
DRICUi066-A	EOAD	F	45	71	33	2.47	stored	on request[b]
DRICUi067-A	EOAD	M	50	69	33	1.64	stored	on request[b]
DRICUi068-A	EOAD	F	50	57	33	1.64	stored	on request[a]
DRICUi069-A	EOAD	F	53	59	34	2.68	stored	on request[b]
DRICUi070-A	EOAD	M	54	58	33	2.35	stored	on request[a]

(*Continued on next page*)

Table 1. *Continued*

Cell line ID	Status	Sex	AAO	AAI	APOE ε-alleles	AD PRS	Fresh/stored	Availability
DRICUi071-A	EOAD	F	53	55	33	2.27	stored	on request[b]
DRICUi072-A	EOAD	M	47	54	33	2.22	stored	on request[a]
DRICUi073-A	EOAD	F	54	59	34	2.41	stored	on request[b]
DRICUi074-A	EOAD	M	51	56	34	2.38	stored	on request[b]
DRICUi075-A	EOAD	F	54	59	33	2.19	stored	on request[b]
DRICUi076-A	control	M	N/A	65	33	−1.88	stored	on request[a]
DRICUi077-A	EOAD	M	45	60	33	2.21	stored	on request[b]
DRICUi078-A	EOAD	F	54	56	33	1.53	stored	on request[b]
DRICUi079-A	EOAD	M	53	62	34	2.35	stored	on request[a]
DRICUi080-A	control	F	N/A	78	33	−2.34	stored	on request[b]
DRICUi081-A	control	F	N/A	78	33	−1.82	stored	on request[b]
DRICUi082-A	control	F	N/A	65	33	−1.71	stored	on request[b]
DRICUi083-A	EOAD	M	52	63	33	1.50	stored	on request[a]
DRICUi084-A	EOAD	M	49	57	33	2.07	stored	on request[a]
DRICUi085-A	EOAD	M	54	56	33	2.07	stored	on request[a]
DRICUi086-A	EOAD	F	50	58	34	2.19	stored	on request[a]
DRICUi087-A	EOAD	M	53	58	34	2.12	stored	on request[a]
DRICUi088-A	control	F	N/A	72	33	−1.81	stored	on request[a]
DRICUi089-A	control	F	N/A	78	33	−2.14	stored	on request[a]
DRICUi090-A	control	F	N/A	88	33	−1.97	stored	on request[a]
DRICUi091-A	control	F	N/A	76	33	−1.98	stored	on request[b]
DRICUi092-A	control	M	N/A	77	33	−2.23	fresh	on request[a]

F, female; M, male; AAO, age at onset; AAI, age at interview (used as a proxy for date of blood collection, first interview used for "stored" blood, and last interview used for "fresh" blood); APOE, apolipoprotein E; AD PRS, global AD polygenic risk score. Fresh indicates that iPSC were derived post 2021 following fresh blood collections from donors. Stored indicates that iPSC were obtained from PBMCs extracted from donor blood prior to 2021 and stored in liquid nitrogen. Availability: iPSC lines that can be obtained by purchase from the EBiSC biobank are labeled "EBiSC," and iPSC lines that can only be obtained from Cardiff University on request are labeled "on request."
[a]iPSC lines are undergoing QC and are anticipated to be available in December 2025 or earlier.
[b]iPSC lines are undergoing QC and are anticipated to be available in February 2026.

as a reference line to demonstrate quality control (QC) assays used on the iPSC.

Establishing the basic cellular identity and pluripotency of the generated iPSC

DRICUi011-A (Figure 1A) was shown to have expected iPSC-like morphology (Figure 1B), to have cleared Cytotune virus components (Figure 1C), and were shown to be free from mycoplasma (Figure S1A). Moreover, iPSC displayed the expected karyotype when compared to the originating expanded T cells (Figures 2 and S1B). Additionally, iPSC were shown to express the pluripotency markers TRA-1-60 and NANOG (Figure 3A) and to successfully differentiate into all three embryonic germ layers (Figure 3B).

DISCUSSION

Most AD research uses iPSC models with individual protein-coding mutations, from familial or common AD-associated risk genes (Penney et al., 2020). While these studies allow us to develop our understanding of the pathways involved in AD pathogenesis, single-gene mutation models

Table 2. Cell line donor information for complement PRS iPSC

DRICU ID	Status	Sex	AAO	AAI	APOE ε-alleles	Complement PRS	Fresh/stored	Availability
DRICUi093-A	LOAD	M	75	85	33	2.23	stored	on request[a]
DRICUi094-A	LOAD	F	82	86	33	2.97	stored	on request[a]
DRICUi095-A	control	F	N/A	70	33	−1.81	stored	on request[a]
DRICUi096-A	control	F	N/A	72	33	−1.73	stored	on request[a]
DRICUi097-A	LOAD	M	69	72	33	2.28	stored	on request[a]
DRICUi098-A	LOAD	F	75	76	33	2.16	stored	on request[a]
DRICUi099-A	LOAD	F	66	69	33	2.53	stored	on request[a]
DRICUi100-A	control	M	N/A	85	33	−2.15	stored	on request[a]
DRICUi101-A	control	F	N/A	82	33	−2.19	stored	on request[a]
DRICUi102-A	control	F	N/A	83	33	−1.81	stored	on request[a]
DRICUi103-A	LOAD	F	73	78	33	2.45	stored	on request[a]
DRICUi104-A	LOAD	F	71	74	33	2.48	stored	on request[a]
DRICUi105-A	control	F	N/A	67	33	−1.77	stored	on request[a]
DRICUi106-A	control	F	N/A	85	33	−1.93	stored	on request[a]
DRICUi107-A	LOAD	F	68	73	33	2.08	stored	on request[a]
DRICUi108-A	LOAD	F	60	67	33	2.37	stored	on request[a]
DRICUi109-A	control	F	N/A	82	33	−2.06	stored	on request[a]
DRICUi110-A	control	M	N/A	77	33	−1.85	stored	on request[a]
DRICUi111-A	control	M	N/A	78	33	−1.55	stored	on request[a]

F, female; M, male; AAO, age at onset; AAI, age at interview (used as a proxy for date of blood collection, first interview used for "stored" blood, and last interview used for "fresh" blood); APOE, apolipoprotein E; complement PRS, complement pathway-specific polygenic risk score. Fresh indicates that iPSC were derived post 2021 following fresh blood collections from donors. Stored indicates that iPSC were obtained from PBMCs extracted from donor blood prior to 2021 and stored in liquid nitrogen. Availability: iPSC lines that can only be obtained from Cardiff University on request are labeled "on request."
[a]iPSC lines are undergoing QC and are expected to be available in February 2026.

do not represent the polygenic diversity seen in common AD individuals and hence do not capture the complexity of disease (Sims et al., 2020). In contrast, the iPSC resource IPMAR represents a major new resource to capture high polygenic risk for common AD within diagnosed individuals. Following differentiation of these iPSC into AD-relevant cell types, researchers will be able to determine how high PRS AD lines differ phenotypically from low PRS control lines. This could be achieved through sequencing techniques, staining, or functional assays. Any differences observed may help identify cellular phenotypes that either contribute to or protect against AD development—phenotypes that are more prevalent in high PRS AD lines may point to factors promoting disease, while those more common in low PRS control lines may suggest protective traits. To start, researchers could focus on differentiating these iPSC into known AD-relevant cell types—such as neurons, astrocytes, and microglia—and examine key AD-related changes, such as microglial inflammatory activation. Furthermore, the iPSC platform could be used during the development of AD therapeutics to model cell dysfunction present in common AD. Where possible, analyses should stratify by APOE genotype to take into account the potential effect of APOE4 on phenotypes observed. Researchers may wish to consider the effect of sex, age, and any heterogeneity of clinical phenotype as potential confounding variables. Future research could also compare EOAD to LOAD iPSC, to explore whether the age of onset correlates with phenotypic severity or whether different phenotypes are enriched in EOAD versus LOAD.

The limitations of the resource include that only White Caucasian subjects from the UK were used; therefore, any translation of research findings will need to take into account that they may not be generalizable to other

A

Cell line ID	Disease status	Sex	AAO	AAI	APOE genotype	AD PRS
DRICUi011-A	LOAD	M	70	80	APOE3/3	2.66

B C

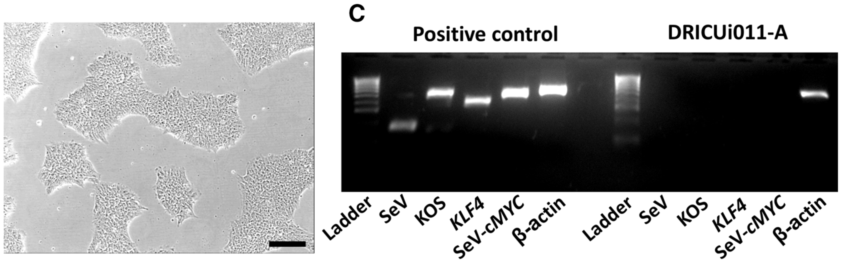

Figure 1. iPSC generated from IPMAR clone DRICUi011-A show expected morphology and are negative for Sendai vector transgenes

(A) Details regarding the IPMAR line featured in this paper, with human pluripotent stem cell registration (hPSC) name DRICUi011-A.

(B) Representative light microscopy image showing colonies of DRICUi011-A induced pluripotent stem cells (iPSC), scale bar: 100 μm.

(C) Cytotune Sendai viral vector components are absent from DRICUi011-A iPSC, shown via comparison to positive control RNA. Product sizes: Sendai virus (SeV) = 181 base pairs (bp), KOS (Krüppel-like factor 4 [*KLF4*], octamer-binding transcription factor 4 [*OCT4*], and sex-determining region Y-box 2 [*SOX2*]) = 528 bp, *KLF4* = 410 bp, SeV-*c-MYC* = 532 bp, β-actin [*ACTB*] = 623 bp. β-actin, a housekeeping gene present in both samples, acts as a positive control. LOAD, late-onset Alzheimer's disease; *APOE*, apolipoprotein E; M, male; AAO, age at onset; AAI, age at interview.

populations and ethnicities. Furthermore, the use of expanded T cells as source material for the iPSC means that T cell receptor regions 7q34 (~0.5 Mb region) and 14q11.2 (~0.7 Mb region) have total or partial deletion in most iPSC, due to T cell receptor recombination events in T cells; therefore, we do not recommend the use of these iPSC for generating T cells, without careful characterization of the T cell receptor repertoire of the lines. iPSC-derived brain cells are also relatively immature *in vitro* and do not preserve the epigenetic profiles of primary cells caused by aging or environmental factors.

PRSs have demonstrated effective application in identifying genetic risk for AD by demonstrating associations between SNPs and AD risk. However, current SNP-based genotyping approaches, such as SNP arrays, struggle with mapping repetitive genomic regions and identifying large structural variants. Long-read sequencing technologies, such as PacBio HiFi and Oxford Nanopore, address these issues effectively but are currently not cost-effective for large-scale studies. As these technologies improve and become more affordable, they will enhance PRS calculations by providing more comprehensive genetic variant detection. Furthermore, it is important to highlight that PRS must be used alongside other clinical factors, such as environmental risk, when ascertaining an overall risk for AD. However, the well-established PRS approach is an effective metric for capturing polygenic risk of AD.

Future iPSC lines developed will include those with high endocytic pathway-specific risk, which will follow the same generation methodologies and QC to allow integration with the current cohort. Given the importance of human and common AD-specific models when investigating the pathogenesis of AD, the IPMAR resource has the potential to revolutionize both AD modeling and drug screening. We enthusiastically welcome collaboration and are eager to share our innovative resource with potential partners.

METHODS

Cohort details

The donors were chosen from participants within the ADCC, which were recruited between 2001 and 2020 using Medical Research Council, Moondance Foundation, and Health and Care Research Wales (HCRW) funding. The present study was approved by the research ethics committee Wales REC 3 (REC ID 12/WA/0052). PBMCs were collected with informed consent under four studies with REC IDs 12/WA/0052, 04/9/030, 17/SS/0139, and 00/09/42. Donors were self-reported Caucasian of European ancestry. The cohort collection used a standardized clinical and comprehensive neuropsychological assessment to diagnose either EOAD or LOAD (see supplemental information). Informed consent was obtained from participants following assessment of capacity to consent (see supplemental information) All AD cases met criteria for either probable (National Institute of Neurological Disorders and Stroke–Alzheimer Disease and Related Disorders (McKhann et al., 1984), Diagnostic and Statistical Manual of Mental Disorders, fourth edition (Association, 2000)) or definite (Consortium to Establish a Registry for Alzheimer's Disease (Rossetti et al., 2010)) AD. All elderly controls were screened for dementia and were chosen to match case samples for sex and ethnicity. Anonymized detailed clinical, cognitive, and non-cognitive longitudinal data and genome-wide microarray data (Illumina 610 or global screening array) from the selected donors will be available upon application via the Dementia Platforms UK website (https://portal.dementiasplatform.uk).

A **Karyogram: PBMC donor of DRICUi011-A**

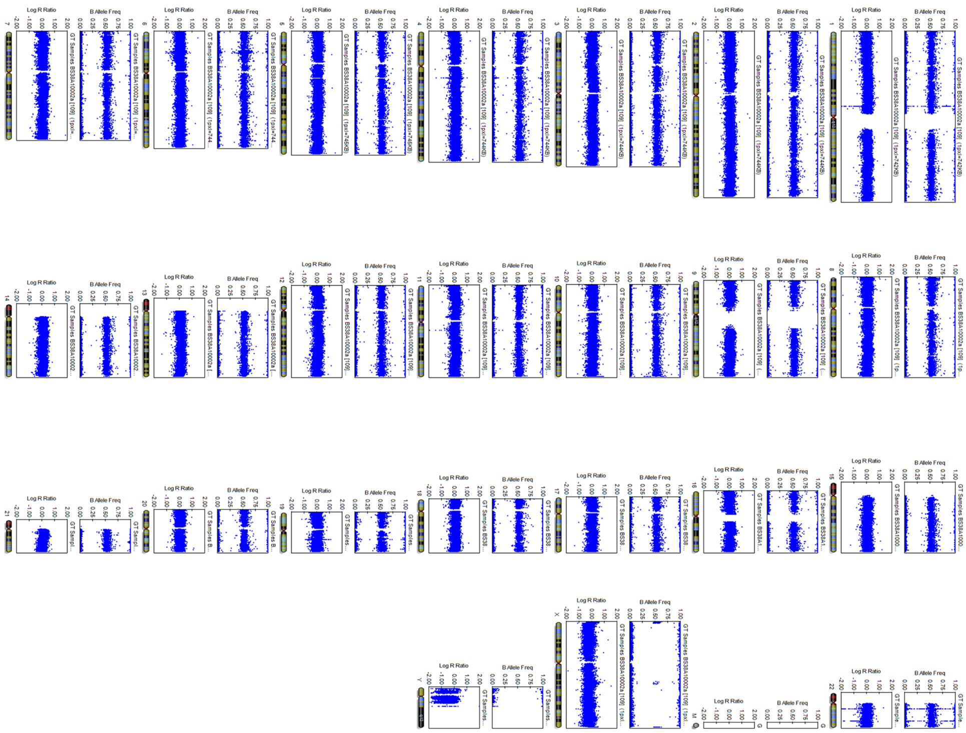

B **Karyogram: iPSC DRICUi011-A**

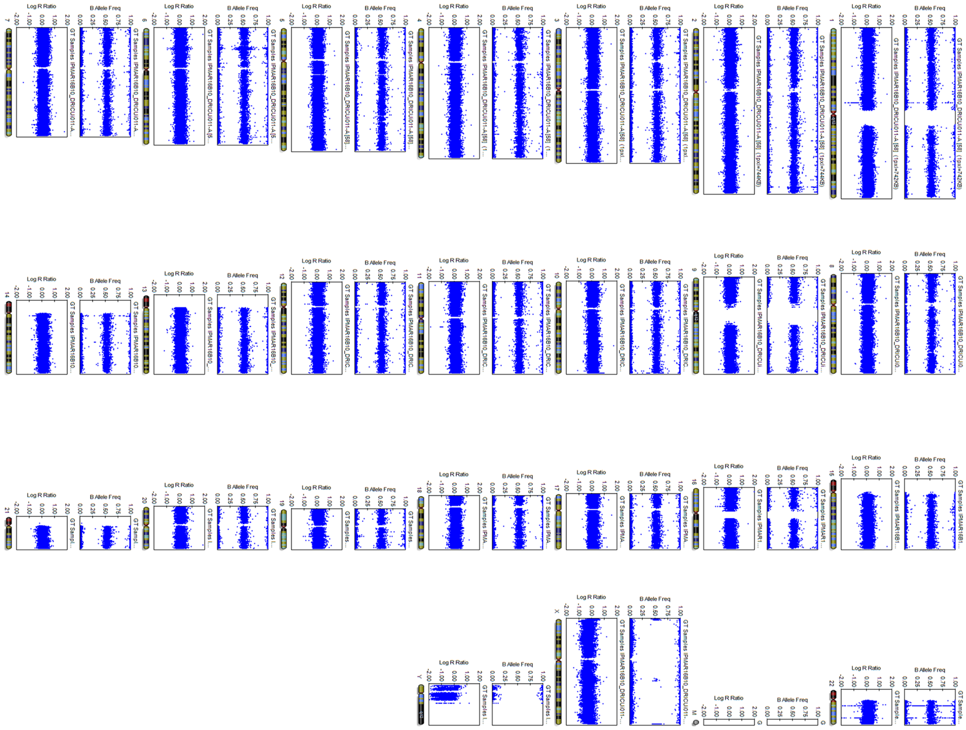

(legend on next page)

Information on ensuring data privacy can be found in the supplemental information.

PRS analysis and donor selection

Global AD PRS lines

Quality control of genetic data was performed before conducting the AD PRS analysis on the ADCC, as detailed in the supplemental methods. Following PRS analysis, individuals were selected from within the cohort that who met one of the following criteria:

(1) LOAD diagnosed, age of onset >65, and with a high LOAD PRS (mainly >1.8 SD).
(2) EOAD diagnosed, age of onset <58, and with a high LOAD PRS (>1.5 SD).
(3) Cognitively normal, *APOE* ε3/3, over the age of 70, and with a low LOAD PRS (mainly <−1.8 SD).

During selection, a balance of male and female donors was aimed for. Additionally, donors with *APOE ε3/3* genotype were prioritized for the LOAD and EOAD lines. All cognitively normal donors were *APOE* ε3/3. Information on individuals selected is provided in Table 1.

Complement PRS lines

Calculation of base and complement PRS was performed using GWAS statistics from the current largest clinically assessed AD case-control cohort (Bellenguez et al., 2022), using the PRSice-2 software package (https://doi.org/10.1093/gigascience/giz082). The default *p* value threshold was selected, and clumping was performed at a linkage disequilibrium (LD) threshold of below 0.1 (r^2 correlation coefficient) within a window of 1 kb to retain weakly correlated variants and remove redundant effects of significant SNPs within high LD. Custom gene matrix transposed (.gmt) files were created containing the complement gene list defined by Carpanini et al. (2021) with loci from the Ensembl/HAVANA merged gene annotation gene transfer format (.gtf) file for human genome build GRCh37.87. SNPs were extracted within the complement gene loci with surrounding windows (−35 kb upstream, +10 kb downstream) to capture intergenic SNPs with potential *cis*-regulatory effects. Raw complement PRSs were adjusted based on the 8 most significant genomic principal components to control for potential population stratification in our samples and then normalized against mean and standard deviation of PRS of non-AD-affected individuals from the 1958 National Child Development cohort (*N* = 4,032) (University College London, UCL Social Research Institute, 2024) to obtain a PRS weighted against the background AD risk of the general population.

After AD-associated complement PRS calculation, individuals were selected based on the following criteria.

(1) LOAD diagnosed, age of onset >65 years, *APOE* ε3/3 with a high complement PRS (>2 SD).
(2) Cognitively normal, *APOE* ε3/3 with a low complement PRS (<−1.5 SD).

Information on individuals selected to generate complement PRS lines is provided in Table 2.

Generation of iPSC

PBMC preparation for recent donations

A subset of the PBMC was obtained following fresh blood collections from donors, obtained post 2021. For fresh blood donations (indicated "fresh" in Table 1), blood was collected in 3 × 6 mL anti-citrate dextrose (ACD) solution B tubes per patient, and PBMCs were extracted using Lymphoprep reagent in SepMate50 tubes, following the manufacturer's protocol (STEMCELL Technologies, 85460). PBMCs were frozen in cryogenic tubes with 1–2 million cells per vial in freeze media containing 90% (v/v) embryonic stem cell-qualified fetal bovine serum (FBS, Fisher, 11500526) and 10% (v/v) dimethyl sulfoxide (DMSO, Merck, D2650) and stored in liquid nitrogen.

Historical PBMC preparation

A subset of the PBMC was extracted from donor blood prior to 2021 (indicated "stored" in Table 1). For these PBMCs, samples were collected in ACD specimen collection tubes (minimum volume 5 mL). The samples were processed using either Accuspin tubes (Sigma, A2055) or Histopaque tubes (Sigma, A7054) when Accuspin tubes were unavailable.

(1) For Accuspin tubes: initially, the blood was poured into Accuspin tubes, with the use of multiple tubes per sample as needed and subjected to centrifugation at 1,000 xg for 25 min at room temperature (RT).
(2) For Histopaque tubes: up to 10 mL of blood was carefully layered onto 5 mL of Histopaque, taking care to

Figure 2. iPSC generated from IPMAR clone DRICUi011-A show expected karyotype

SNP array to test for chromosomal aberrations in gene-edited iPSC clones. For each chromosome, the first dot plot displays the B-allele frequency and indicates whether the SNV (single-nucleotide variant) is heterozygous (data points fall at around 0.5) or homozygous (data point at around 0.0 of 1.0). The second plot that displays the log R value (first dot plot, indicated with R on the left side) is given, representing the probe intensity of individual SNVs. Chromosomal gains are identified by doubling of the log R value while halving the R value indicates loss.

(A) Karyograms from the original PBMC sample and (B) from the derived DRICUi011-A iPSC.

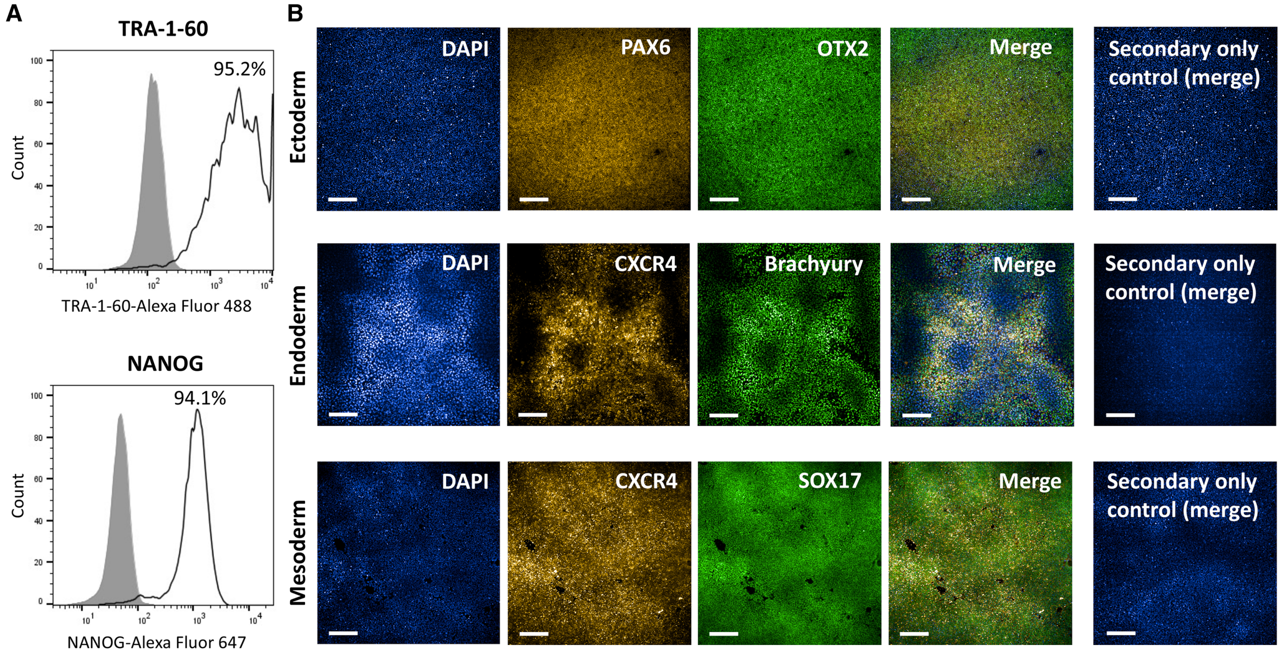

Figure 3. iPSC generated from IPMAR clone DRICUi011-A express pluripotency markers and are able to differentiate into all three germ layers

(A) Flow cytometric analysis of DRICUi011-A iPSC demonstrates the expression of pluripotency markers TRA-1-60 and NANOG. Bold line indicates antigen-specific staining, and gray histograms denote isotype control staining.

(B) Following trilineage differentiation of DRICUi011-A iPSC, expression of the ectodermal markers OTX2 (green) and PAX6 (red), mesodermal markers brachyury (green) and CXCR4 (red), and endodermal markers SOX17 (green) and CXCR4 (red) was demonstrated. A secondary antibody-only staining control (including DAPI) is shown with merged channels for each germ layer. Scale bars: 200 μm.

avoid mixing of phases, followed by centrifugation at 1,000 xg for 20 min. For samples with less than 2 mL, microtubes containing 0.5 mL of Histopaque were used, with 1 mL of blood added to each tube and centrifuged at 4,500 xg for 10 min.

Subsequently, if the separation of the white cell layer from red blood cells was insufficient, the sample was centrifuged again. The supernatant above the frit was then transferred into a freshly labeled Accuspin (or Histopaque) tube and centrifuged at 1,000 xg for 20 min at 20 ± 1°C. The resultant white, cloudy layer was isolated between the filter and clear yellow serum layer and transferred to a fresh 15 mL tube. Following this, up to 10 mL of pre-warmed RPMI 1640 (without serum, Fisher, 11875093) was added to the sample, centrifuged at 250 xg for 10 min at RT. After discarding the supernatant, the pellet was resuspended in pre-warmed RPMI (without serum) and centrifuged again at 250 xg for 10 min at RT. The size of the pellet was assessed, and based on this and the quality of separation, the decision to store 1 or 2 ampoules was made. Generally, 2 ampoules were made from 7 to 10 mL blood and only 1 from 4 to 5 mL. Samples intended for freezing as PBMCs were resuspended in 1 mL of freeze media (90% (v/v) FBS +10% (v/v) DMSO) per 1.8 mL cryotube used and frozen using a Kryo 10 rate controlled freezer before transferring to permanent long-term storage in liquid nitrogen.

iPSC reprogramming

T cells were expanded from PBMCs prior to iPSC reprogramming, by thawing PBMCs and culturing in RPMI 1640 media (Thermo Fisher Scientific, 12004997) supplemented with 10% (v/v) FBS (Fisher, 11500526) and 35 ng/mL interleukin (IL)-2 (Merck, SRP3085), in 12-well plates coated with 10 μg/mL CD3 (OKT3) monoclonal antibody (Thermo Fisher Scientific, 15276737). T cells were initially expanded for 7–10 days and were frozen in freeze media containing 90% (v/v) embryonic stem cell-qualified FBS (Fisher, 11500526) and 10% (v/v) DMSO (Merck, D2650) and stored in liquid nitrogen. Three days before reprogramming T cells were cultured in OpTmizer CTS (Gibco, A1022-01) medium with 10% embryonic stem cell grade FBS (Gibco, 16141-079), 2 mM Glutamax (Gibco, 35030-01), and 35 ng/mL IL-2 (Sigma, SRP6170) and activated with beads (ratio 1:2 or 1:1) coated with CD2, CD3, and CD28 (T cell activation expansion kit, Miltenyi Biotec, 130-091-441).

For LOAD samples (IDs up to DRICUi055-A), 300,000 cells per donor were reprogrammed using the Cytotune-iPS

2.0 Sendai Reprogramming kit (Thermo Fisher Scientific, A16517) at multiplicity of infection 5 following the manufacturer's protocol for feeder-dependent iPSC reprogramming of fibroblasts. Vector transduced cells were transferred onto mitotically inactivated CF1 Mouse Embryonic Feeder cells (Millipore, PMEF-CFL-C) on 0.1% gelatin (Sigma, G1393)-coated plates. From day 3, cells were cultured in Knock-Out serum replacement medium (Knock-out DMEM [Gibco, 10829-018], 20% KO serum replacement [Gibco, 10828-028], 2 mM Glutamax [Gibco, 35030-1], 1% non-essential amino acids [Gibco, 11140-035], 100 units/mL penicillin and 100 μg/mL streptomycin [Gibco, 15140-122], 55 μM 2-mercaptoethanol [Gibco, 31350-010], and 5 ng/mL bFGF [Miltenyi Biotec, 130-093-842]). Daily 50% medium changes were carried out, and from day 10, MEF-conditioned medium was used. Colonies with iPSC morphology were manually picked on approximately day 20 and transferred to Geltrex (Gibco, A14133-02)-coated wells with mTeSR-1 medium (STEMCELL Technologies, 85850) and 10 μM ROCK inhibitor Y-27632 (Abcam, ab120129), with daily 100% medium changes performed henceforth. iPSC lines were passaged every 5–7 days using 0.5 mM EDTA (Life Technologies, 15575-038) in PBS (Sigma, D8537) to lift and replate clumps of cells into fresh Geltrex-coated plates in mTeSR-1 or mTeSR Plus (STEMCELL Technologies, 100-0276) medium without ROCK inhibitor. Cells were frozen at passage 10 in freeze medium containing 10% (v/v) DMSO, 30% (v/v) embryonic stem cell grade FBS (Gibco, 16141-079), and 60% (v/v) Knock-out DMEM (Gibco, 10829-018) while checking clearance of Sendai vectors by PCR and were then thawed for further passage/checking if not clear, or for expansion if clear. Expansion was carried out over a minimum number of passages, and approximately 30 vials (2 million cells per vial) master stock at P12–20 were frozen per line, in the same freeze medium used at passage 10. Bulk frozen stocks were tested for mycoplasma, and the QC assays described in the following section were performed.

For EOAD and complement PRS iPSC (IDs upward from DRCUi056-A), donor-expanded T cells were reprogrammed to iPSC by Oxford StemTech Ltd. using a proprietary Sendai vector-based method with their ReproPlex platform.

QC of iPSC

Real-time PCR to confirm clearance of Cytotune Sendai vectors

RNA was extracted from approximately 1.6 million cells by RNeasy Mini Kit (QIAGEN, 74004) at p10. 1 μg of RNA was reverse transcribed using a RetroScript kit (Ambion, 10585595) or the RevertAid kit (Thermo Scientific, K1622), and this was then diluted 1:5 in sterile water. A 20 μL PCR reaction of 10 μL AmpliTaq gold DNA polymerase (Applied Biosystems, 4398881), 0.5 μL of 10 μM forward primer and 0.5 μL of 10 μM reverse primer (primer pairs shown in Table S1), 2 μL cDNA, and 7 μL sterile water was used to amplify genes present on the viral vectors. The PCR products were run on a 1.5% agarose TAE gel with a 100 bp ladder (NEB, N3231S) and imaged using Bio-Rad ChemiDoc XRS+ and Bio-Rad Image Lab software. Viral vector components checked included Sendai virus, Krüppel-like factor 4 (*KLF4*), Sendai virus-*c*-MYC, and KOS, which is an acronym used for the combination of genes *KLF4*, octamer-binding transcription factor 3/4, and sex-determining region Y-box 2. A β-actin control was also run. If viral vectors were not cleared, bands would be visible for all markers; if the viral vectors had cleared, only a β-actin control band would be present. Positive controls, generated from T cells 3 days post infection, were always run in parallel to samples.

Flow cytometry for pluripotency markers

Pluripotency of iPSC was assessed in the bulk-frozen iPSC using flow cytometry for pluripotency markers TRA-1-60 and NANOG (see Table S2 for details of antibodies and isotype controls used), with appropriate isotype controls, using the same concentration and supplier. Cells were fixed for 10 min in 2% (w/v) paraformaldehyde (PFA) in PBS (Alfa Aesar) and permeabilized in 100% methanol at −20°C for at least 30 min before staining. For antibody staining, methanol was removed from the cells, and they were subsequently washed twice in staining buffer (0.1% (w/v) bovine serum albumin in PBS). 50,000 cells were used for each condition: blank, TRA-1-60 isotype control, TRA-1-60, NANOG isotype control, and NANOG. Samples were incubated with antibodies for 45 min at RT, in the dark, and with gentle shaking. Cells were washed twice and kept on ice during flow cytometry analysis. Measurement was conducted using FACS Calibur (Becton Dickinson) or Attune NxT (Thermo Fisher Scientific) flow cytometers, with analysis using FlowJo.

Immunocytochemistry staining for trilineage markers

Trilineage differentiation was performed on all generated iPSC lines to confirm their pluripotency and ability to differentiate to all 3 germ layers (ectoderm, endoderm, and mesoderm). Differentiation was performed in 24-well plates according to manufacturer's instructions (STEMdiff Trilineage Differentiation Kit, STEMCELL Technologies, 05230). Following differentiation, cells were fixed with 4% PFA (w/v) (10 min at RT) and washed twice with PBS. Fixed cells were incubated with blocking buffer (1% [w/v] BSA, 10% [v/v] Normal Donkey Serum, and 0.3% [v/v] Triton X-100 in PBS) for 1 hour at RT prior to overnight incubation at 4°C with primary antibodies diluted in blocking buffer. Antibodies against OTX2 and PAX6 were used to confirm ectoderm identity, antibodies against brachyury and CXCR4 for mesoderm, and SOX17 and CXCR4 for endoderm (details of primary antibodies used can be found in Table S3). Following overnight incubation, cells were

washed three times with PBS prior to a 1-hour incubation at RT in the dark with fluorescent secondary antibodies diluted in PBS plus NucBlue Live ReadyProbes Reagent (Fisher, R37605). Secondary antibodies used were donkey anti-mouse Alexa Fluor Plus 555 (Fisher, 15970296), donkey anti-rabbit Alexa Fluor 568 (Fisher, 10617183), and donkey anti-goat Alexa Fluor Plus 488 (Fisher, 15930877). Cells were then washed twice with PBS prior to imaging at 10X on an Opera Phenix high-content screen system (PerkinElmer).

SNP Copy-number variant analysis

SNP copy-number variant (CNV) analysis was performed on DNA from both the bulk-frozen iPSC and the original expanded donor T cells. DNA was extracted from approximately 1.6 million cells using DNeasy blood and tissue kit (QIAGEN, 69504). 15 μL of 100 ng/μL was used for SNP CNV array, using Illumina GSA-24v3-0_A1. The SNP CNV array was performed either by Life & Brain GmbH (Germany), or in-house at the Cardiff University School of Medicine. Illumina Genome Studio 2.0 software was used to analyze the data and produce karyograms and correlation plots; PennCNV was used for detailed CNV calling. Abnormalities over 1 million base pairs were considered to fail QC, using log R ratio and B allele frequency, and furthermore any size of 20q duplication. Abnormalities over 0.4 million base pairs will be reported with the cell line record on https://hPSCreg.eu. Alignment scatterplot with parent sample was also carried out with R^2 to confirm the relationship between derived line and parent.

Short tandem repeat profiling

DNA from both the bulk-frozen iPSC and the original expanded donor T cells underwent short tandem repeat (STR) profiling analysis. DNA was extracted from approximately 1.6 million cells using the DNeasy blood and tissue kit (QIAGEN, 69504). Following extraction, STR profiling was carried out by Northgene (UK).

Post thaw viability, morphology, and mycoplasma assessment

iPSC stock vials were thawed to assess the morphology and viability of frozen stocks, as well as to confirm the absence of mycoplasma and microbial contaminants. One vial of approximately 2 million cells was thawed, and cells were counted using Chemometec Nucleo counter NC-3000. Cells were distributed between two Geltrex (Gibco, A14100-02)-coated wells at 80% and 20% densities, respectively. After 48 h, images were acquired using AMG Evos XL core digital inverted microscope at 10× magnification, and cells were inspected at high magnification to confirm the absence of bacteria or fungus. For the mycoplasma testing, either Mycoalert was used (Lonza, LT07-418) following manufacturer's instructions, a ratio of 0–0.999 was considered negative, or cell supernatants were tested for mycoplasma by Eurofins Genomics UK Ltd.

RESOURCE AVAILABILITY

Lead contact

- Reasonable requests for further information and resources should be directed to and will be fulfilled by the lead contact, Hazel Hall-Roberts (hall-robertsh@cardiff.ac.uk).

Materials availability

- All iPSC generated in this study will be made available on request to bona fide researchers for a specified peer-reviewed research project. iPSC will be supplied under a materials transfer agreement and for a compensation fee paid by the requestor for stock maintenance and shipping. As the iPSC were generated using CytoTune technology, they are subject to the CytoTune limited use label license (see supplemental information), which restricts commercial use to holders of the label license. Consent provisions and study ethics permit commercial use and animal use of the iPSC.
- Some iPSC generated in this study will additionally be made available via the European Bank for iPSC EBiSC (www.ebisc.org), as indicated in Table 1.
- At the time of publication, some iPSC lines are not distribution-ready, as indicated in Tables 1 and 2; however, the authors welcome requests and will communicate timescales to interested parties.

Data and code availability

- QC data for the iPSC are available on https://hPSCreg.eu for lines that are distribution-ready and will be released for the remaining lines following the timescales indicated in Tables 1 and 2.
- Anonymized detailed clinical, cognitive, and non-cognitive longitudinal data and genome-wide microarray data (Illumina 610 or global screening array) from the donors used in this study are deposited with the Dementias Platform UK Data Portal (https://portal.dementiasplatform.uk) and are publicly available, subject to approval by a data access committee and a completed data access agreement. Information on which cohorts to request access and the donor IDs for each iPSC line is provided by the Cell Line Discovery tool (https://portal.dementiasplatform.uk/ipmar/cell-line-discovery-tool/) on the Dementias Platform UK Data Portal.
- This paper does not report original code.
- Any additional information required to reanalyze the data reported in this paper is available from the lead contact upon reasonable request.

ACKNOWLEDGMENTS

Collection of the ADCC patient cohort was supported by the Medical Research Council (MRC) Center (MR/L010305/1 and MR/T04604X/1), the UK Dementia Research Institute (UKDRI supported by the Medical Research Council [MRC] [UKDRI-3003], Alzheimer's Research UK, and Alzheimer's Society), Welsh Government, Joint Programming for Neurodegeneration (JPND), and the Moondance Foundation. The global AD PRS iPSC were

generated with funding from the UK Dementia Research Institute (award number UK DRI-3201) through UK DRI Ltd, principally funded by the Medical Research Council. The complement PRS iPSC were generated with funding from the Moondance Foundation and Alzheimer's Research UK. The James and Lillian Martin Center for Stem Cell Research, Oxford, is supported by the James Martin 21st Century Research Foundation, and reprogramming utilized capital equipment funded by National Institute for Health Research-Medical Research Council Dementias Platform UK Equipment Award (MR/M024962/1).

The authors would like to thank the families and individuals who donated samples and participated in the study for their invaluable contributions. The authors would like to thank Oxford StemTech for the reprogramming of the EOAD and complement PRS iPSC lines (IDs upward from DRICUi056-A) and Core Technical Team from the Cardiff University Center for Neuropsychiatric Genetics and Genomics for performing SNP CNV arrays on most of the iPSC lines. Additionally, the authors would like to thank the EBiSC biobank for hosting the IPMAR LOAD iPSC platform, and Dementias Platform UK for developing a discovery tool for the data associated with the IPMAR iPSC and hosting the associated dataset.

AUTHOR CONTRIBUTIONS

J. Williams, N.D.A., V.E.-P., C.W., P.R.T., R.S., S.A.C., and G.L. conceived and designed the global AD PRS iPSC project. E.S. advised on the reprogramming strategy. H.H.-R. managed the global AD PRS iPSC project. S.M.C. and J. Williams conceived and designed the complement PRS iPSC project. S.M.C. managed the complement PRS iPSC project. A.C.M. and S.K. calculated PRSs and aided with donor selection. A.E. analyzed the SNP CNV data. R.S., N.D., and J. Williams managed the human sample collections. R.M., L.L., and N.D. performed fresh blood draws and administration for the human sample collections. J. Winston and R.O. processed fresh bloods. J. Winston and S.M.C. expanded donor T cells for reprogramming. S.H.E. and L.B. reprogrammed iPSC for the global PRS LOAD lines. S.H.E., J. Winston, E.M., R.O., and B.S. cultured iPSC and performed QC assays. E.M. and H.H.-R. wrote the manuscript.

DECLARATION OF INTERESTS

S.A.C. and S.H.E. receive research funding from GSK, and S.A.C. has received funding from Eli Lilly and Janssen.

SUPPLEMENTAL INFORMATION

Supplemental information can be found online at https://doi.org/10.1016/j.stemcr.2025.102570.

Received: November 15, 2024
Revised: June 4, 2025
Accepted: June 5, 2025
Published: July 3, 2025

REFERENCES

Al Abbar, A., Ngai, S.C., Nograles, N., Alhaji, S.Y., and Abdullah, S. (2020). Induced Pluripotent Stem Cells: Reprogramming Platforms and Applications in Cell Replacement Therapy. Biores. Open Access *9*, 121–136. https://doi.org/10.1089/BIORES.2019.0046.

Association, A.P. (2000). Diagnostic and Statistical Manual of Mental Disorders Fourth Edition Text Revision (DSM-IV-TR) (American Psychiatric Association).

Bellenguez, C., Küçükali, F., Jansen, I.E., Kleineidam, L., Moreno-Grau, S., Amin, N., Naj, A.C., Campos-Martin, R., Grenier-Boley, B., Andrade, V., et al. (2022). New insights into the genetic etiology of Alzheimer's disease and related dementias. Nat. Genet. *54*, 412–436. https://doi.org/10.1038/s41588-022-01024-z.

Belloy, M.E., Andrews, S.J., Le Guen, Y., Cuccaro, M., Farrer, L.A., Napolioni, V., and Greicius, M.D. (2023). APOE Genotype and Alzheimer Disease Risk Across Age, Sex, and Population Ancestry. JAMA Neurol. *80*, 1284–1294. https://doi.org/10.1001/JAMANEUROL.2023.3599.

Carpanini, S.M., Harwood, J.C., Baker, E., Torvell, M., Sims, R., Williams, J., and Morgan, B.P. (2021). The Impact of Complement Genes on the Risk of Late-Onset Alzheimer's Disease. Genes *12*, 443. https://doi.org/10.3390/genes12030443.

Drummond, E., and Wisniewski, T. (2017). Alzheimer's Disease: Experimental Models and Reality. Acta Neuropathol. *133*, 155–175. https://doi.org/10.1007/S00401-016-1662-X.

Escott-Price, V., Myers, A.J., Huentelman, M., and Hardy, J. (2017). Polygenic Risk Score Analysis of Pathologically Confirmed Alzheimer's Disease. Ann. Neurol. *82*, 311–314. https://doi.org/10.1002/ANA.24999.

Franco Bocanegra, D.K., Nicoll, J.A.R., and Boche, D. (2018). Innate immunity in Alzheimer's disease: the relevance of animal models? J. Neural Transm. *125*, 827–846. https://doi.org/10.1007/S00702-017-1729-4.

Ganesan, K., Rentsch, P., Langdon, A., Milham, L.T., and Vissel, B. (2024). Modeling sporadic Alzheimer's disease in mice by combining Apolipoprotein E4 risk gene with environmental risk factors. Front. Aging Neurosci. *16*, 1357405. https://doi.org/10.3389/FNAGI.2024.1357405.

Kondo, T., Hara, N., Koyama, S., Yada, Y., Tsukita, K., Nagahashi, A., Ikeuchi, T., Ishii, K., Asada, T., Arai, T., et al. (2022). Dissection of the polygenic architecture of neuronal Aβ production using a large sample of individual iPSC lines derived from Alzheimer's disease patients. Nat. Aging *2*, 125–139. https://doi.org/10.1038/s43587-021-00158-9.

Kukull, W.A., Schellenberg, G.D., Bowen, J.D., McCormick, W.C., Yu, C.-E., Teri, L., Thompson, J.D., O'Meara, E.S., and Larson, E.B. (1996). Apolipoprotein E in Alzheimer's disease risk and case detection: A case-control study. J. Clin. Epidemiol. *49*, 1143–1148. https://doi.org/10.1016/0895-4356(96)00195-3.

Lagomarsino, V.N., Pearse, R.V., Liu, L., Hsieh, Y.C., Fernandez, M.A., Vinton, E.A., Paull, D., Felsky, D., Tasaki, S., Gaiteri, C., et al. (2021). Stem cell derived neurons reflect features of protein networks, neuropathology and cognitive outcome of their aged human donors. Neuron *109*, 3402–3420.e9. https://doi.org/10.1016/J.NEURON.2021.08.003.

Mancuso, R., Van Den Daele, J., Fattorelli, N., Wolfs, L., Balusu, S., Burton, O., Liston, A., Sierksma, A., Fourne, Y., Poovathingal, S., et al. (2019). Stem-cell-derived human microglia transplanted in

mouse brain to study human disease. Nat. Neurosci. *22*, 2111–2116. https://doi.org/10.1038/s41593-019-0525-x.

McKean, N.E., Handley, R.R., and Snell, R.G. (2021). A Review of the Current Mammalian Models of Alzheimer's Disease and Challenges That Need to Be Overcome. Int. J. Mol. Sci. *22*, 13168. https://doi.org/10.3390/IJMS222313168.

McKhann, G., Drachman, D., Folstein, M., Katzman, R., Price, D., and Stadlan, E.M. (1984). Clinical diagnosis of Alzheimer's disease. Neurology *34*, 939–944. https://doi.org/10.1212/WNL.34.7.939.

Penney, J., Ralvenius, W.T., and Tsai, L.H. (2020). Modeling Alzheimer's disease with iPSC-derived brain cells. Mol. Psychiatr. *25*, 148–167. https://doi.org/10.1038/S41380-019-0468-3.

Raska, J., Hribkova, H., Klimova, H., Fedorova, V., Barak, M., Barta, T., Pospisilova, V., Vochyanova, S., Vanova, T., and Bohaciakova, D. (2021). Generation of six human iPSC lines from patients with a familial Alzheimer's disease (n = 3) and sex- and age-matched healthy controls (n = 3). Stem Cell Res. *53*, 102379. https://doi.org/10.1016/j.scr.2021.102379.

Reitz, C., Rogaeva, E., and Beecham, G.W. (2020). Late-onset vs nonmendelian early-onset Alzheimer disease: A distinction without a difference? Neurol. Genet. *6*, e512. https://doi.org/10.1212/NXG.0000000000000512.

Ringman, J.M., Monsell, S., Ng, D.W., Zhou, Y., Nguyen, A., Coppola, G., Van Berlo, V., Mendez, M.F., Tung, S., Weintraub, S., et al. (2016). Neuropathology of autosomal dominant Alzheimer disease in the National Alzheimer Coordinating Center database. J. Neuropathol. Exp. Neurol. *75*, 284–290. https://doi.org/10.1093/jnen/nlv028.

Rossetti, H.C., Cullum, C.M., Hynan, L.S., and Lacritz, L.H. (2010). The CERAD Neuropsychological Battery Total Score and the Progression of Alzheimer's Disease. Alzheimer Dis. Assoc. Disord. *24*, 138. https://doi.org/10.1097/WAD.0B013E3181B76415.

Saddiki, H., Fayosse, A., Cognat, E., Sabia, S., Engelborghs, S., Wallon, D., Alexopoulos, P., Blennow, K., Zetterberg, H., Parnetti, L., et al. (2020). Age and the association between apolipoprotein E genotype and Alzheimer disease: A cerebrospinal fluid biomarker–based case–control study. PLoS Med. *17*, e1003289. https://doi.org/10.1371/JOURNAL.PMED.1003289.

Serrano-Pozo, A., Frosch, M.P., Masliah, E., and Hyman, B.T. (2011). Neuropathological Alterations in Alzheimer Disease. Cold Spring Harb. Perspect. Med. *1*, a006189. https://doi.org/10.1101/CSHPERSPECT.A006189.

Sims, R., Hill, M., and Williams, J. (2020). The multiplex model of the genetics of Alzheimer's disease. Nat. Neurosci. *23*, 311–322. https://doi.org/10.1038/s41593-020-0599-5.

Tran, K.M., Kawauchi, S., Kramár, E.A., Rezaie, N., Liang, H.Y., Sakr, J.S., Gomez-Arboledas, A., Arreola, M.A., Cunha, C.D., Phan, J., et al. (2023). A Trem2R47H mouse model without cryptic splicing drives age- and disease-dependent tissue damage and synaptic loss in response to plaques. Mol. Neurodegener. *18*, 12. https://doi.org/10.1186/S13024-023-00598-4.

Wingo, T.S., Lah, J.J., Levey, A.I., and Cutler, D.J. (2012). Autosomal Recessive Causes Likely in Early-Onset Alzheimer Disease. Arch. Neurol. *69*, 59–64. https://doi.org/10.1001/ARCHNEUROL.2011.221.

Stem Cell Reports, Volume *20*

Supplemental Information

Modeling common Alzheimer's disease with high and low polygenic risk in human iPSC: A large-scale research resource

Emily Maguire, Jincy Winston, Sarah H. Ellwood, Rachel O'Donoghue, Bethany Shaw, Atahualpa Castillo Morales, Samuel Keat, Alexandra Evans, Rachel Marshall, Lauren Luckcuck, Laura Brown, Elisa Salis, Ganna Leonenko, Nicola Denning, EADB consortium, Nicholas D. Allen, Valentina Escott-Price, Caleb Webber, Philip R. Taylor, Rebecca Sims, Sally A. Cowley, Julie Williams, Sarah M. Carpanini, and Hazel Hall-Roberts

Supplemental figures

A Mycoplasma

Sample	Reading 1	Reading 2	Ratio	Status
Positive control	3.113	137.3	44.1	Positive
Negative control	4.135	0.713	0.17	Negative
DRICUi011-A	2.048	0.953	0.47	Negative

B CNV correlation between DRICUi011-A iPSC and donor PBMC

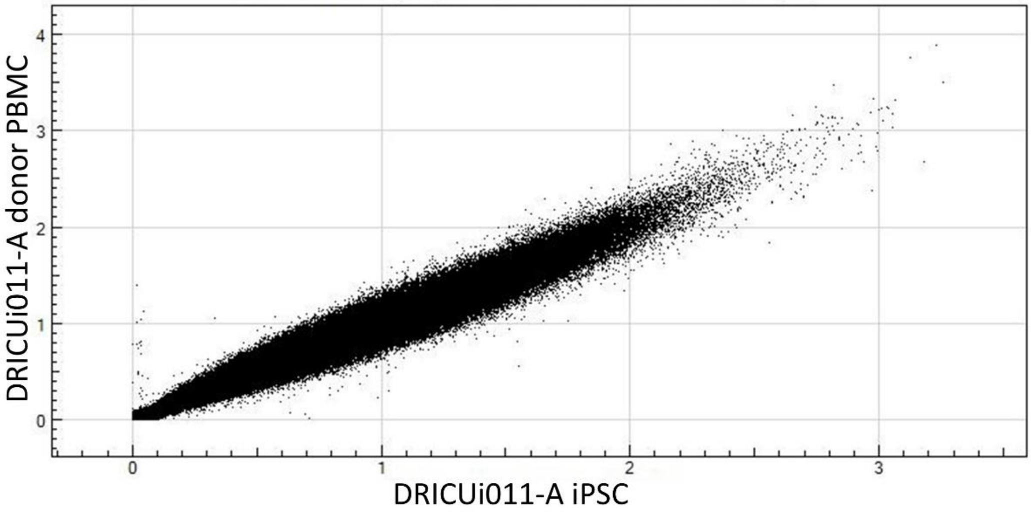

Figure S1. A) DRICUi011-A iPSC test negative for mycoplasma at passage 14, ratios between 0-0.999 indicate a negative result (green), ratios above 1.3 indicate a positive result (red). B) Copy number variant analysis shows a strong correlation between iPSC-line DRICUi011-A and the donor PBMC for that line, regression coefficient R^2= 0.98.

Supplemental tables

Table S1: Primers used to identify clearance of Sendai virus.

Marker	Forward primer	Reverse primer	Product size
**SeV**	GGATCACTAGGTGATATCGAG	ACCAGACAAGAGTTTAAGAGATATGTATC	181bp
**KOS**	ATGCACCGCTACGACGTGAGCGC	ACCTTGACAATCCTGATGTGG	528bp
**KLF4**	TTCCTGCATGCCAGAGGAGCCC	AATGTATCGAAGGTGCTCAA	410bp
**SeV-Myc**	AACTGACTAGCAGGCTTGTCG	TCCACATACAGTCCTGGATGATGATG	532bp
**β-Actin**	AGGCATCCTCACCCTGAAG	TCCATGCCCAGGAAGGAAG	632bp

SeV = Sendai virus, KLF4 = Krüppel-like factor 4 (Klf4), KOS = KLF4, Octamer-binding transcription factor 3/4 (Oct3/4), & Sex determining region Y-box 2 (Sox2), bp = base pairs.

Table S2: Details regarding antibodies used for flow cytometry of pluripotency markers.

Antibody	Supplier	Catalogue number	Stock concentration	Staining concentration
**Alexa Fluor® 488 Mouse IgM, κ Isotype control**	Biolegend	401617	500 μg/mL	3 μg/mL
**Alexa Fluor® 488 anti-human TRA-1-60-R**	Biolegend	330614	150 μg/mL	3 μg/mL
**Rabbit (DA1E) mAb IgG XP® Isotype Control (Alexa Fluor® 647 Conjugate)**	Cell Signaling Technology	2985S	100 μg/mL	0.67 μg/mL
**Nanog (D73G4) XP® Rabbit mAb (Alexa Fluor® 647 Conjugate)**	Cell Signaling Technology	5448S	50 μg/mL	0.67 μg/mL

Table S3: Details regarding antibodies used for immunocytochemistry staining for trilineage markers.

Germ Layer	Antibody	Code/ Company	Against	Stock concentration	Staining concentration
**Ectoderm**	OTX2	AF1979	Goat	0.2 mg/mL	10 μg/mL
	PAX6	901302	Rabbit	2 mg/mL	40 μg/mL
**Mesoderm**	Brachyury	AF2085	Goat	0.2 mg/mL	10 μg/mL
	CXCR4	MAB172	Mouse	0.5 mg/mL	8 μg/mL
**Endoderm**	SOX17	AF1924	Goat	0.2 mg/mL	10 μg/mL
	CXCR4	MAB172	Mouse	0.5 mg/mL	8 μg/mL

Supplemental methods

Cohort assessment for AD

The collection of the samples was through multiple channels, including specialist NHS services and clinics, research registers and Join Dementia Research (JDR) platform. The participants were assessed at home or in research clinics along with an informant, usually a spouse, family member or close friend, who provided information about and on behalf of the individual with dementia. Established measures, validated for AD by Holmes et al., [1] were used to ascertain clinical diagnosis and the disease severity: CAMDEX, Mattis Dementia Rating Scale, Mini-Mental state examination (MMSE), Bristol activities of daily living (BADL), Clinical Dementia Rating scale (CDR), Neuropsychiatric Inventory (NPI), Global Deterioration Scale (GiDS), Addenbrooke's Cognitive Examination (ACE-r), Geriatric Depression Scale (GeDS) and National Adult Reading Test (NART). All AD cases met criteria for either probable (NINCDS-ADRDA, DSM-IV) or definite (CERAD) AD. Control participants were recruited from GP surgeries and by means of self-referral. All elderly controls were screened for dementia using the Mini Mental State Examination (MMSE) or ADAS-cog. Control samples were chosen to match case samples for sex and ethnicity.

Informed consent for AD subjects

Capacity to consent was assessed by a trained, experienced research assistant at all visits. In the case where an individual lacked capacity to consent, an appropriate consultee was identified in accordance with section 32 of the Mental Capacity Act (2005). All participants and consultees received verbal and written information about the study and had the opportunity to ask questions. Participation was voluntary, with the option to withdraw consent at any time, without giving a reason. Any signs of the participant objecting or feeling distressed during a visit were assumed as refusal of consent, leading to cessation of sample/data collection.

Data privacy

The confidentiality of participants in this study is preserved in accordance with the Data Protection Act (1998). All participant information is stored in a linked pseudo-anonymous format, with participants assigned an ID code. Personal identifying information linked to the ID code is stored separately from the clinical and genetic data in a password-protected spreadsheet, within Cardiff University's data security infrastructure, and can only be accessed by designated team members.

Quality control of Genetic data

Our PRS based prioritisation of cell lines was based on genotyping data derived from multiple DRI Cardiff collections. Genotyping of carried out in different stages and funded by MRC and CADR grant applications.

a) GERAD dataset (3332 cases and 7355 controls) was genotyped on the Illumina550 array (GRCh37/hg19), with Quality-Control (QC) analysis described in previously [2]. Further it was imputed via Michigan Imputation server using Minimac3 [3] and Haplotype Reference Consortium (HRC)3 reference panel. After imputation QC: variants were excluded with minor allele frequency (MAF) <1%, missing data proportion (MISS) >5% and Hardy-Weinberg Equilibrium (HWE) ($p \leq 10^{-6}$) and poor imputation INFO score of <0.7. Illumina550 array did not have APOE gene and APOE genotyping was done separately.

b) EADB 2 batches were genotyped on Illumina GSA (GRCh37/hg19) array in Lille (5679 samples and 678,826 SNPs. Sample QC: exclude gender mismatch (164 individuals); duplicates in name (3 individuals), het >+-0.1 (11 individuals); population stratification (42 individuals). Variant QC: missingness >2%, MAF<1%, (HWE) ($p \leq 10^{-6}$). Leaving 5283 samples on 491611 samples. APOE genotype was performed separately. This dataset was part of EADB consortium.

c) 1909 individuals were genotyped on Illumina GSA array (GRCh37/hg19) covering 665,608 variants. APOE genotype was included as part of the array. This data is independent from IGAP and EADB consortiums.

Datasets b) and c) were combined together and additional quality control performed to exclude samples with high heterozygosity (Het $\pm$ 0.05); individual missingness > 3%; high identity by descent (pi^hat > 0.2) or gender mismatch. Variants with MAF < 1%; missingness > 5%; and that break Hardy-Weinberg Equilibrium (HEW $p \leq 10^{-6}$). Furthermore, control-control analysis was performed and variants were excluded with $p<5x10^{-3}$, leaving 6,870 samples and 480,021 variants. This combined data set was then imputed using Michigan Imputation server's Minimac3[3] and Haplotype Reference Consortium (HRC)3 reference panel. Following imputation, variants with minor allele frequency (MAF) <1%, missing data proportion (MISS) >5% and Hardy-Weinberg Equilibrium (HWE) ($p \leq 10^{-6}$) and poor imputation INFO score of <0.7 were excluded, leaving 7,518246 polymorphisms. Finally, all the datasets were combined together with genotyping data from non-AD affected individuals from the 1958 National Child Development (N= 4,032) [4] as a population control, for the purposes of PRS standardisation and principal components were re-calculated.

Primary PRS calculation (C + T)

For the PRS calculation we used the summary statistics from the largest available clinically assessed case-control GWAS study on AD [5] ($N = 63{,}926$) to generate genetic scores for all participants in the cohorts described above as the weighted sum of the risk alleles. PRS were generated using PRSice-2 using default P-value thresholds. PRSice-2 utilizes the most common approach for PRS calculation of clumping and thresholding (C + T), where markers most strongly associated with the phenotype of interest are preferentially retained. Clumping was performed using an LD r^2=0.1 and a window size of 1000 kb. PRS.no.APOE was calculated excluding the *APOE* region (chromosome 19:44.4–46.5 Mb) due to the high LD in this region. PRS.AD was calculated as a weighted sum of PRS.no.APOE and *APOE*(ε2 + ε4), where *APOE* effects were weighted with effect sizes (B(ε2) = −0.47 and B(ε4) = 1.12) [6]. This means that, for APOE ε3 homozygous individuals, their PRS.AD is equivalent to their PRS.no.APOE. PRS.AD was then adjusted by regressing against 8 principal components and then standardised within the sample. Subsequently, PRS.AD of all of the individuals in our cohorts were standardised against the mean and standard deviation of the PRS.AD of the population controls in 1958BC.

Commercial use

Consent provisions and study ethics permit commercial use of the iPSC and related data. However, the iPSC were generated using CytoTune technology and are subject to the terms of Limited use label License No: 518 CytoTune™ Technology for Products, as detailed below.

Notice to Purchaser: This product is authorized for reprogramming methods that involve or pertain to the preparation of iPS cells or related cells. The purchase of this product conveys to the purchaser the limited, non-transferable right to use the purchased amount of product to perform internal use and for educational purposes. No right to resell this product or any of its components, or iPS cells or related cells generated by use of the product, or derivatives thereof (hereafter "the Materials") is conveyed expressly, by implication, or by estoppel. For clarity, purchasers have the right to use third party service providers for generating iPS cells and derivatives for the benefit of such purchasers. Purchasers can deposit the Materials with not-for-profit repositories ("Repositories") and transfer cells to not-for-profit research entities (not affiliated with a for-profit organization) for their internal research. Such recipient Repositories and not-for-profit research entities are allowed to distribute the Materials not-for financial gain to other users for their internal research. If the Materials are transferred to other users in accordance with the terms of this label license accompanying the product (hereafter "Label License"), the transferring party should notify recipients of such Materials of these terms by transferring a copy of the Label License to the recipients.

To obtain commercial rights for the sale of the Materials or for a fee-for-service generation of the Materials other than as allowed in paragraph 1 above, purchasers are requested to contact DNAVEC Corporation at cytotune@dnavec-corp.com. For all other commercial applications relating to the use of the Materials, purchasers might be required to contact iPS Academia Japan. Customers may contact iPS Academia Japan either directly at license@ips-ac.co.jp or through DNAVEC Corporation.

Supplemental references

1. Holmes, C., Cairns, N., Lantos, P. L. & Mann, A. Validity of current clinical criteria for Alzheimer's disease, vascular dementia and dementia with Lewy bodies. *British Journal of Psychiatry* **174**, 45–50 (1999).

2. Harold, D. *et al.* Genome-wide association study identifies variants at CLU and PICALM associated with Alzheimer's disease. *Nat Genet* **41**, 1088–1093 (2009).

3. Das, S. *et al.* Next-generation genotype imputation service and methods. *Nat Genet* **48**, 1284–1287 (2016).

4. University College London, UCL Social Research Institute, C. for L. S. National Child Development Study [data series]. *UK Data Service 14th release* (2024) doi:http://doi.org/10.5255/UKDA-Series-2000032.

5. Kunkle, B. W. *et al.* Genetic meta-analysis of diagnosed Alzheimer's disease identifies new risk loci and implicates Aβ, tau, immunity and lipid processing. *Nat Genet* **51**, 414–430 (2019).

6. Leonenko, G. *et al.* Identifying individuals with high risk of Alzheimer's disease using polygenic risk scores. *Nat Commun* **12**, 4506 (2021).
